# Priority actions to fight antibiotic resistance: results of an international meeting

**DOI:** 10.1186/2047-2994-1-17

**Published:** 2012-05-03

**Authors:** Vincent Jarlier, Jean Carlet, John McGowan, Herman Goossens, Andreas Voss, Stephan Harbarth, Didier Pittet

**Affiliations:** 1 UPMC Univ Paris 6 EA1541 Bactériologie-Hygiène 75005 and Hôpital Pitié-Salpêtrière, Assistance Publique, Hôpitaux de Paris, 75013, France; 2 Consultant, WHO African Partnerships for Patient Safety, 9 rue de la Terrasse, 94000, Créteil, France; 3 Department of Epidemiology, Rollins School of Public Health of Emory University, Atlanta, GA, 30322, USA; 4 Microbiology Laboratory, University Hospital Antwerp, Wilrijkstraat 10, 2650, Edegem, Belgium; 5 Canisius-Wilhelmina Ziekenhuis and Radboud University Nijmegen Medical Centre, Postbus 9015, 6500 GS, Nijmegen, The Netherlands; 6 Infection Control Programme and WHO Collaborating Centre on Patient Safety, University of Geneva Hospitals and Faculty of Medicine, 4 Rue Gabrielle-Perret-Gentil, 1211, Geneva 14, Switzerland; 7 Bactériologie-Hygiène Faculté de Médecine P. et M. Curie, 91 Bd de l’Hôpital, 75013, Paris, France

## 

Over 70 international experts in medicine, infectious diseases, microbiology and epidemiology (see list in Additional file 1: Annex 1), coming from 33 countries, met from June 27–29 June 2011 in Annecy *(France)* for the third edition of the World HAI Forum on healthcare-associated infections. The aim of this meeting was to release a global call to action to fight antibiotic resistance. While most meetings focus on scientific developments retrospectively in an academic format, World HAI Forums, which are held every two years, gives participating experts a chance to do prospective analysis of subjects that are not usually discussed [1]. A large part of the time is devoted to sharing best practices, successes and failures in the fight against health care associated infections and resistant bacteria, as a basis for building effective action plans.

The 3^rd^ world HAI forum was entirely dedicated to the challenge represented by the increase in antibiotic resistance. Indeed, while research to discover novel antibiotics has slowed to a virtual standstill, bacterial resistance has increased due (a) to the massive use and misuse of antibiotics, not only for human health, but also for animals and (b) to the cross transmission of resistant bacteria in the health care setting and in the community due to insufficient levels of hygiene. The treatment of certain common infections is becoming difficult because of antibiotic resistance and, concurrently, the success of immunosuppressive therapies and surgical interventions (organ transplants, cardiac surgery…), which are associated with a high risk of bacterial infection due to resulting host compromise.

To the Forum experts, the current emergence in Europe of pan-resistant NDM-1 bacteria and the epidemic of multidrug-resistant *E. coli* infections should be taken as a major public health warning, indicating that a new era of antimicrobial resistance has begun. This must lead to a global awakening: the protection of antibiotics has now entered the sphere of sustainable development.

Forum participants (33 countries indicated above) presented and discussed programs for surveying and controlling health care associated infections and antibiotic resistance. These programs and related success stories were mainly presented as posters (see list in Additional file 2: Annex 2, with links to actual posters) whose content was thoroughly discussed during several specific sessions. These posters also were displayed during the 1^st^ International Conference on Prevention and Infection Control (ICPIC) held in Geneva in July 2011.

The content of these posters, and the main results obtained following the implementation of these programs, has been summarized thereafter in a world map (Figure 1). For that purpose, they have been classified in five categories depending on their scope: (a) Antibiotic stewardship and infection control, (b) Antibiotic stewardship, (c) Infection control, (d) Survey (antibiotic consumption, bacterial resistance to antibiotics, health care associated infections and (e) International action. These programs have in many places led to a decrease in antibiotic consumption and antibiotic resistance (*e.g.* in organisms such as MRSA, VRE, carbapenem resistant enterobacteria, and *Pseudomonas aeruginosa*).

**Figure 1:**
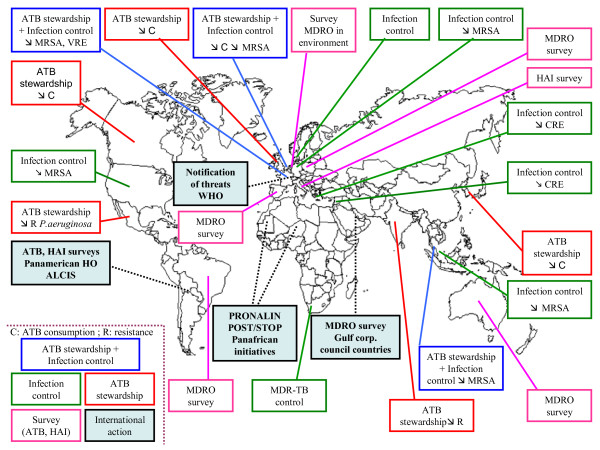
**Summary of the content and results of national and international programs for surveying and controlling health care associated infections and antibiotic resistance, presented as posters at the 3**^**rd**^**world HAI forum.** The posters have been classified in five categories depending on their scope: (a) Antibiotic stewardship and infection control, (b) Antibiotic stewardship, (c) Infection control, (d) Survey (antibiotic consumption, bacterial resistance to antibiotics, health care associated infections and (e) International action. Symbols used: ↘ = diminution; C = antibiotic consumption; MRSA = methicillin resistant S*taphylococcus aureus*; VRE = vancomycin resistant enterococci; CRE = carbapenem resistant enterobacteria; MDRO = multidrug resistant organisms; R = bacterial resistance.

In a continuation of calls to action and proposals made by major national and international organizations (WHO, ECDC, IDSA, CDC, etc.), and in addition to a collective position paper [2], the Forum’s participants (see list in Additional file 1: Annex 1) voted to rank by priority 24 messages to prevent an impending public health catastrophe caused by the emergence and spread of bacteria that are resistant to all antibiotics. The ranking of these messages was organized in 4 groups corresponding to distinct categories of stakeholders: national and international health authorities and policy makers, the medical and veterinary communities, the general public, and Industry. The results of the ranking are displayed in Additional file 3: Annex 3.

Based on the results of the vote, the 24 messages have been synthesized to be easily presented as 12 actions to be implemented in the short to mid-term, as follows.

## Priority actions for policy makers and health authorities

For animals, stop the administration of antibiotics used in human medicine and limit antibiotics to therapeutic use only. It is imperative to reserve the most important classes of antibiotics for humans.

Banish, in all countries, the use of antibiotics as growth promoters in animal feed.

Regulate the sale of antibiotics for use in human medicine and prohibit over-the-counter sales worldwide.

Have international organizations (WHO, European Union) develop a charter on good antibiotic stewardship and have all the ministries of health worldwide sign it and commit to respecting it.

## Priority actions for the human and veterinary healthcare communities

Establish standardized, universal surveillance of antibiotic use and resistance and monitor the emergence and spread of new forms of bacterial resistance.

Include, in medical and veterinary school curricula, a solid training in bacterial resistance and the prudent use of antibiotics and establish on-the-job training programs for healthcare workers, taking into account the cultural specificities of each country.

## Priority actions for the general public

Develop culturally sensitive awareness campaigns, targeted to the general public, explaining the importance of protecting antibiotics and using them only when absolutely necessary.

Provide education about fundamental hygiene, such as handwashing, to prevent the spread of infection. It is imperative to improve sanitation systems to eliminate resistant bacteria in wastewater.

Include consumers in the development and implementation of action plans.

## Priority actions for industry

Develop point-of-care and rapid diagnostic tests, which can be used at the patient’s bedside or in the doctor’s office, to guide the prescription of antibiotics and avoid their prescription for viral infections.

Stimulate research and development of novel antibiotics.

Find new economic models which reconcile public health interests with Industry needs for profitability.

## Supplementary Material

Additional file 1 **Annex1 - Participants in the 3**^**rd**^**World HAI Forum.** Click here for file

Additional file 2 **Annex 2 - List of posters presenting national and international programs for surveying and controlling health care associated infections and antibiotic resistance at the 3**^**rd**^world HAI forum.* Click here for file

Additional file 3 **Annex 3 - Result of the vote organized during the 3**^**rd**^**world HAI forum to rank by priority 24 actions to prevent an impending public health catastrophe caused by the emergence and spread of bacteria that are resistant to all antibiotics.** The ranking was organized in 4 groups corresponding to distinct categories of stakeholders. The actions are ranked from priorities 1 to 6 (or 7) and the corresponding ballots (voting: all forum attendees see Additional file 1: Annex 1). Click here for file
