# Supplementary material for: Priority actions to fight antibiotic resistance: results of an international meeting
Source: Antimicrob Resist Infect Control. 2012 May 3;1:17. doi: 10.1186/2047-2994-1-17 (PMC3436666; doi:10.1186/2047-2994-1-17)
Supplement: Additional file 1 — Annex1 - Participants in the 3rdWorld HAI Forum. [file 2047-2994-1-17-S1.doc]

**Annex 1: Participants in the 3^rd^ World HAI Forum**

ABDUL GHAFUR Kulakkattil, Chennai, India

ALLEGRANZI Benedetta, Geneva , Switzerland

AWANG JALIL Nordiah, Kuala Lumpur, Malaysia.

BALKHY Hanan, Riyadh, Saudi Arabia

BAVESTRELLO Luis, Viña del Mar, Chile

CANTON Rafael, Madrid, Spain

CARLET Jean, Créteil, France

CARMELI Yehuda, Tel Aviv, Israel

COIGNARD Bruno, Saint Maurice, France

CONA Erna, Santiago de Chile, Chile

CONLY John, Calgary, Canada

COOKSON Barry, London,United Kingdom

CORSO Alejandra, Buenos Aires, Argentina

CREMIEUX Anne Claude, Garches, France

CYRILLO Marcos Antonio, Sao Paulo, Brazil

DURLACH Ricardo Augusto, Buenos Aires, Argentina

DUSE Adriano, Houghton, South Africa

FANNING Seamus, Dublin, Ireland

GASTMEIER Petra, Berlin, Germany

GOLDMANN Donald, Boston, USA

GOOSSENS Herman, Antwerp, Belgium

GOTTLIEB Thomas, Woollahra, Australia

GRAYSON Lindsay, Victoria, Australia

GUZMAN BLANCO Manuel, Caracas, Venezuela

HARBARTH Stephan, Geneva, Switzerland

HAUSTEIN Thomas, Geneva, Switzerland

HERWALDT Loreen, Iowa, USA

HOLLIS Aidan, Calgary, Canada

HOLMES Alison, London, United Kingdom

HOSOGLU Salih, Diyarbakir, Turkey

HRYNIEWICZ Waleria, Warsaw, Poland

HU Bijie, Shanghai, China

JAGGI Namita, Gurgaon-Haryana, India

JARLIER Vincent, Paris, France

JARVIS William, Port Orford, USA

KAKU Mitsuo, Sendai, Japan

KIM Eui-Chong, Seoul, Korea

KLUGMAN Keith, Atlanta, USA

KLUYTMANS Jan, Breda, The Netherlands

LING Moi Lin, Singapore

MARTINEZ-MARTINEZ Luis, Santander, Spain

McGOWAN John, Atlanta, USA

MEHTAR Shaheen, Cape Town, South Africa

MORO Maria Luisa, Bologna, Italy

NATHWANI Dilip, Dundee, United Kingdom

NDOYE Babacar, Dakar, Senegal

NICOLLE Lindsay, Winnipeg, Canada

NORDMANN Patrice, Le-Kremlin-Bicetre, France

PATERSON David, Bulimba, Australia.

PERENCEVICH Eli, Iowa City, USA

PERL Trish, Baltimore, USA

PITTET Didier, Geneva, Switzerland

RICHTMANN Rosanna, São Paulo, Brazil

ROSSI Flavia, São Paulo, Brazil

RYAN John, European Commission, Luxembourg

SAMORE Mattew, Utah, USA

SETO Wing Hong, Hong Kong, SAR, China

SIFUENTES OSORNIO José, Mexico City, Mexico

SKOV Robert, Copenhagen, Denmark

SOMOGYI PEREZ Teresa, San Jose, Costa Rica

SRINIVASAN Arjun, Atlanta, USA

TACCONELLI Evelina, Rome, Italy

TSAKRIS Athanassios, Athens, Greece

UPHAM Garance, Previssin, France

VALLEJO Martha, Medellin, Columbia

VAN BELKUM Alex, La Balme Les Grottes, France

VANDENBROUCKE-GRAULS Christina, Amsterdam, The Netherlands

VANHEMS Philippe, Lyon, France

VOSS Andreas, Nijmegen, The Netherlands

WALSH Timothy, Cardiff, United Kingdom
