# Supplementary material for: Priority actions to fight antibiotic resistance: results of an international meeting
Source: Antimicrob Resist Infect Control. 2012 May 3;1:17. doi: 10.1186/2047-2994-1-17 (PMC3436666; doi:10.1186/2047-2994-1-17)
Supplement: Additional file 2 — Annex 2 - List of posters presenting national and international programs for surveying and controlling health care associated infections and antibiotic resistance at the 3rdworld HAI forum.* [file 2047-2994-1-17-S2.doc]

**Annex 2: List of posters presenting national and international programs for surveying and controlling health care associated infections and antibiotic resistance at the 3^rd^ world HAI forum***(to access the PDF of the actual poster, click on the title)

| Senegal | Babacar Ndoye | [Infection Control Experience and Challenges in Francophone West Africa](http://www.biomerieux-diagnostics.com/upload/Babacar Ndoye Poster Senegal-1.pdf) |
| --- | --- | --- |
| South Africa | Shaheen Mehtar | [Control of MDR Tuberculosis: protecting patients and healthcare workers in South Africa](http://www.biomerieux-diagnostics.com/upload/Shaheen Mehtar South Africa-1.pdf) |
| Australia | David Paterson | [Multidrug resistant organisms in Australia: Imports and Home-grown](http://www.biomerieux-diagnostics.com/upload/David Paterson Poster Australia-1.pdf) |
| India | Namita Jaggi | [Control of multi resistant bacteria in India](http://www.biomerieux-diagnostics.com/upload/Namita JaggiPoster India-1.pdf) |
| India | Abdul Ghafur | [Save antibiotics, save lives](http://www.biomerieux-diagnostics.com/upload/Abdul Ghafur Poster India-2.pdf) |
| Japan | Mitsuo Kaku | [Control of antimicrobial resistant organisms in Japan](http://www.biomerieux-diagnostics.com/upload/Mitsuo Kaku Poster Japan-1.pdf) |
| South Korea | Eui-Chong Kim | [Antibiotic control in Korea](http://www.biomerieux-diagnostics.com/upload/Eui Chong Kim Poster Korea-1.pdf) |
| Malaysia | Nordiah Jalil | [Challenges of antimicrobial use control in Malaysia](http://www.biomerieux-diagnostics.com/upload/Nordiah Jalil Poster Malaysia-1.pdf) |
| Singapore | Moi Lin Ling | [Impact of a hospital-wide hand hygiene promotion strategy on healthcare associated infections](http://www.biomerieux-diagnostics.com/upload/Moi Lin Ling Poster Singapour-1.pdf) |
| Belgium | Herman Goossens | [Achievements of the Belgian Antibiotic Policy Coordination Committee (BAPCOC)](http://www.biomerieux-diagnostics.com/upload/Herman Goossens Belgium-1.pdf) |
| Israel | Yehuda Carmeli | [Experience of outpatient fluoroquinolone restriction in Israel](http://www.biomerieux-diagnostics.com/upload/Yehuda Carmeli Poster Israel-1.pdf) |
| Denmark | Robert Skov | [Successful control of community acquired MRSA in Denmark: mission impossible?](http://www.biomerieux-diagnostics.com/upload/Robert Skov Poster Denmark-1.pdf) |
| France | Bruno Coignard | [Control of Imported Multi-Drug Resistant Organisms: the French Experience](http://www.biomerieux-diagnostics.com/upload/Bruno Coignard Poster France-1.pdf) |
| France | Vincent Jarlier | [20 years of Antimicrobial Resistance control in a multi hospital institution (Assistance Publique - Hôpitaux de Paris](http://www.biomerieux-diagnostics.com/upload/Vincent Jarlier Poster France-1.pdf)) |
| France | Anne Claude Cremieux | [Impact of the 2 first national Antibiotic plans in France](http://www.biomerieux-diagnostics.com/upload/Anne Claude Cremieux Poster France-1.pdf) |
| Germany | Petra Gastmeier | [Antimicrobial Resistance data from the German KISS system](http://www.biomerieux-diagnostics.com/upload/Petra Gastmeier Poster Germany-1.pdf) |
| Greece | Athanassios Tsakris | [Imported KPC-producing *Klebsiella pneumoniae* clones in a Greek hospital: infection control measures to restrain their dissemination](http://www.biomerieux-diagnostics.com/upload/Athanassios Tsakris Poster Greece-1.pdf) |
| Italy | Maria Luisa Moro | [Infection Control and multi resistance in Long Term Care Facilities in Europe](http://www.biomerieux-diagnostics.com/upload/Maria Luisa Moro Poster Europe Italy-1.pdf) |
| Netherlands | Christina Vandenbroucke | [The Netherlands: can we reconcile veterinarian and human antibiotic usage?](http://www.biomerieux-diagnostics.com/upload/Chritina Vandebroucke Poster Netherlands-1.pdf) |
| Poland | Waleria Hryniewicz | [Control of carbapenemases spread in Poland at the regional and country level. Early stage of dissemination of KPC producing *Enterobacteriaceae* in Poland (2007-2008)](http://www.biomerieux-diagnostics.com/upload/Waleria Hryniewicz Poland Poster-1.pdf) |
| Switzerland | Thomas Haustein | [Reliability and Validity Testing of Notification Decisions Under the International Health Regulations (IHR): When are Microbial Threats Considered a Global Concern?](http://www.biomerieux-diagnostics.com/upload/Thomas Haustein Poster WHO-1.pdf) |
| Saudi Arabia | Hanan Balkhy | [The prevalence of antimicrobial resistance in clinical isolates of Gulf Cooperation Council countries and infection control achievements](http://www.biomerieux-diagnostics.com/upload/Hannan Balkhy Revised Poster Saudi Arabia-3.pdf) |
| Spain | Rafael Canton | [The top challenging resistant bacteria in Spain through different surveillance systems](http://www.biomerieux-diagnostics.com/upload/Rafael Canton Poster Spain-2.pdf) |
| UK | Dilip Nathwani | [Antimicrobial Stewardship in Scotland: Impact of a national Programme](http://www.biomerieux-diagnostics.com/upload/Dilip Nathwani Poster Scottland-1.pdf) |
| Argentina | Alejandra Corso | [Latin-American Quality Control Program in Bacteriology and Antibiotic Resistance (OPS): Is the Region prepared to detect emerging mechanisms of resistance](http://www.biomerieux-diagnostics.com/upload/Alejandra Corso Poster Argentina-1.pdf) |
| Brazil | Flavia Rossi | [Trends in bacterial resistance in Brazil](http://www.biomerieux-diagnostics.com/upload/Flavia Ross Poster Brazil-1.pdf) |
| Colombia | Martha Vallejo | [ALCIS project - surveillance network](http://www.biomerieux-diagnostics.com/upload/Martha Vallejo Poster Latin America-1.pdf) |
| Mexico | Jose Sifuentes Osornio | [Antimicrobial utilization and susceptibility patterns of a sentinel group of bacterial isolates prior and subsequent to the introduction of Ertapenem to the hospital formulary](http://www.biomerieux-diagnostics.com/upload/Jose Sifuentes Osornio Poster Mexico-1.pdf) |
| Venezuela | Manuel Guzman | [Spread of VRE and MRSA in Latin America](http://www.biomerieux-diagnostics.com/upload/Manuel Guzman Poster Venezuela-1.pdf) |
| Canada | John Conly | [Antimicrobial Resistance Programs in Canada 1995-2010 : A Critical Evaluation](http://www.biomerieux-diagnostics.com/upload/John Conly Poster Canada-1.pdf) |
| Canada | Lindsay Nicolle | [Control of antimicrobial resistance in Canada: Any lesson to learn?](http://www.biomerieux-diagnostics.com/upload/Lindsay Nicolle Poster Canada-3.pdf) |
| USA | Eli Perencevich | Modeling the future of Antimicrobial Resistance: USA perspective |
| USA | Matthew Samore | [Surveillance and control of MRSA in the VA health care system in the USA](http://www.biomerieux-diagnostics.com/upload/Matthew Samore Poster USA-1.pdf) |
| WHO | Garance Upham | [Patients for patient Safety organization committed to play a key role in combatings antimicrobial reistance in healthcare settings in poor countries of Africa](http://www.biomerieux-diagnostics.com/upload/Garance Upham Poster Africa-1.pdf) |

*** Person listed presented poster at conference; many posters also had co-authors**
