# Supplementary material for: Priority actions to fight antibiotic resistance: results of an international meeting
Source: Antimicrob Resist Infect Control. 2012 May 3;1:17. doi: 10.1186/2047-2994-1-17 (PMC3436666; doi:10.1186/2047-2994-1-17)
Supplement: Additional file 3 — Annex 3 - Result of the vote organized during the 3rdworld HAI forum to rank by priority 24 actions to prevent an impending public health catastrophe caused by the emergence and spread of bacteria that are resistant to all antibiotics. The ranking was organized in 4 groups corresponding to distinct categories of stakeholders. The actions are ranked from priorities 1 to 6 (or 7) and the corresponding ballots (voting: all forum attendees see Additional file 1: Annex 1). [file 2047-2994-1-17-S3.doc]

**Annex 3. Result of the vote organized during the 3^rd^ world HAI forum to rank by priority 24 actions to prevent an impending public health catastrophe caused by the emergence and spread of bacteria that are resistant to all antibiotics. The ranking was organized in 4 groups corresponding to distinct categories of stakeholders. The actions are ranked from priorities 1 to 6 (or 7) and the corresponding ballots (voting: all forum attendees see annex 1).**

**1. What would be your message to Policy Makers or Health Authorities**

1.1. Reserve the most important classes of antibiotics for human use only 66%

1.2. Make a proposal for WHO to develop a chart to be signed by health ministries 51%

1.2. Stop over-the-counter sales of antibiotics 51%

1.4. Change the reimbursement system to ensure that prudent use of antibiotics is rewarded even for pharmaceutical companies 46%

1.5. Make antibiotics a specific category of drugs (add antibiotics to world heritage UNESCO) 31%

1.6. Stop antibiotic sales by doctors and vets 25%

1.7. Develop culturally sensitive awareness campaigns targeted to policy makers 25%

**2. What would be your message to the Healthcare Community**

2.1. Establish standardized, real time, universal surveillance of antibiotic use and resistance 82%

2.2. Educate on antibiotic stewardship and resistance using modern tools (pre and post graduates including veterinary sector) 75%

2.3. Develop culturally sensitive awareness campaigns targeted to healthcare professionals 49%

2.4. Provide public with indicators on Infection Control in healthcare settings 36%

2.5. Standardize antibiotic prophylaxis. Stop abuse (maximum 1 day in surgery) 27%

2.6. Avoid use of quinolones 15%

2.7. Protect carbapenems as the last line antibiotics 15%

**3. What would be your message to the General Public**

3.1. Develop culturally sensitive awareness campaigns targeted to general public 75%

3.2. Develop sanitation and hygiene education 72%

3.3. Include consumers among stakeholders for antibiotic resistance control, including in food chain 51%

3.4. Correct consumer expectations with regard to antibiotic use and healthcare associated infections 48%

3.5. Stop antibiotic self-medication 37%

3.6. Balance costs and benefits of antibiotic-free food 15%

**4. What would be your message to Industry (Pharma, food, diagnostics, farming/bioindustry)?**

4.1. Develop bedside and rapid tests to better guide antibiotic treatment decisions 63%

4.2. Banish the use of antibiotics as growth promoters in animal food 60%

4.3. Develop new antibiotics 57%

4.4. Develop alternatives to antibiotics (eg vaccines) 46%

4.5. Develop responsible marketing 37%

4.6. Improve engineering systems to eliminate resistant bacteria in waste 31%
